# Benzene-1,3,5-tricarb­oxy­lic acid–5-(4-pyrid­yl)pyrimidine (1/1)

**DOI:** 10.1107/S1600536811051075

**Published:** 2011-12-03

**Authors:** Yan-Ke Jiang, Gui-Ge Hou

**Affiliations:** a Research Center of Medical Chemistry & Chemical Biology, Chongqing Technology and Business University, Chongqing 400067, People’s Republic of China; b College of Pharmacy, Binzhou Medical University, Yantai 264003, People’s Republic of China

## Abstract

In the pyrimidine mol­ecule of the title compound, C_9_H_7_N_3_·C_9_H_6_O_6_, the pyridine ring is oriented at 33.26 (11)° with respect to the pyrimidine ring. In the benzene-1,3,5-tricarb­oxy­lic acid mol­ecule, the three carb­oxy groups are twisted by 7.92 (9), 8.68 (10) and 17.07 (10)° relative to the benzene ring. Classical O—H⋯N and O—H⋯O hydrogen bonds and weak C—H⋯O and C—H⋯N hydrogen bonds occur in the crystal structure.

## Related literature

For hydrogen bonding in pyrimidine derivatives, see: Hou *et al.* (2011 ▶); Horikoshi *et al.* (2004 ▶); Georgiev *et al.* (2004 ▶); Santoni *et al.* (2008 ▶); Huang & Parquette (2000 ▶). For co-crystals of organic acids and pyrimidine, see: Bhogala & Nangia(2003 ▶); Du *et al.* (2005 ▶); Hou *et al.* (2008 ▶).
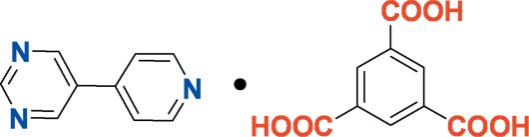


## Experimental

### 

#### Crystal data

C_9_H_7_N_3_·C_9_H_6_O_6_
*M* *_r_* = 367.31
Monoclinic, 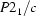

*a* = 8.3532 (19) Å
*b* = 14.865 (3) Å
*c* = 13.066 (3) Å
β = 98.325 (4)°
*V* = 1605.4 (6) Å^3^
*Z* = 4
Mo *K*α radiation
μ = 0.12 mm^−1^
*T* = 298 K
0.32 × 0.12 × 0.10 mm

#### Data collection

Bruker SMART APEX CCD diffractometer
8278 measured reflections
2967 independent reflections
2142 reflections with *I* > 2σ(*I*)
*R*_int_ = 0.042

#### Refinement

*R*[*F* ^2^ > 2σ(*F* ^2^)] = 0.055
*wR*(*F* ^2^) = 0.130
*S* = 1.04
2967 reflections
256 parameters
H atoms treated by a mixture of independent and constrained refinement
Δρ_max_ = 0.22 e Å^−3^
Δρ_min_ = −0.21 e Å^−3^

### 

Data collection: *SMART* (Bruker, 2007 ▶); cell refinement: *SAINT* (Bruker, 2007 ▶); data reduction: *SAINT*; program(s) used to solve structure: *SHELXTL* (Sheldrick, 2008 ▶); program(s) used to refine structure: *SHELXTL*; molecular graphics: *SHELXTL*; software used to prepare material for publication: *SHELXTL*.

## Supplementary Material

Crystal structure: contains datablock(s) global, I. DOI: 10.1107/S1600536811051075/xu5398sup1.cif

Structure factors: contains datablock(s) I. DOI: 10.1107/S1600536811051075/xu5398Isup2.hkl

Supplementary material file. DOI: 10.1107/S1600536811051075/xu5398Isup3.cml

Additional supplementary materials:  crystallographic information; 3D view; checkCIF report

## Figures and Tables

**Table 1:** Hydrogen-bond geometry (Å, °)

*D*—H⋯*A*	*D*—H	H⋯*A*	*D*⋯*A*	*D*—H⋯*A*
O1—H1⋯N3^i^	0.95 (3)	1.70 (3)	2.652 (2)	173 (3)
O3—H3*A*⋯O2^ii^	0.92 (3)	1.84 (3)	2.720 (2)	161 (3)
O5—H5*A*⋯N1	0.95 (3)	1.68 (3)	2.626 (2)	177 (3)
C3—H3⋯O3^ii^	0.93	2.48	3.367 (3)	159
C14—H14⋯N2^iii^	0.93	2.59	3.335 (3)	137

Symmetry codes: (i) 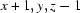; (ii) 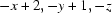; (iii) 
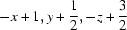
.

**Fig. 1.**
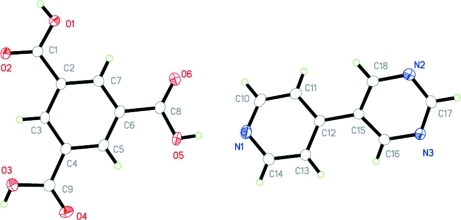
The structure of the title compound with 30% probability displacement ellipsoids.

**Fig. 2.**
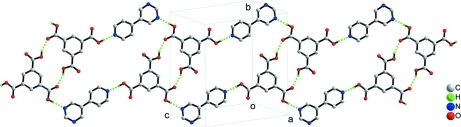
A view of the hydrogen-bonded double-chain observed in the crystal structure of (1).

## supplementary crystallographic information

### Comment

Some pyrimidine derivatives, such as such as 5, 5'-dipyrimidine, 1,2-bis(5'-pyrimidyl)ethyne, 5-(3-pyridyl)pyrimidine (L), 5-phenyl-2-(4-pyrid-yl)pyrimidine, and 4-(pyridin-2-yl)pyrimidine-2-sulfonate, could form strong hydrogen-bond interaction and play an essential role in synthesis of supra-molecular structure (Hou *et al.*, 2011; Horikoshi *et al.*, 2004; Georgiev *et al.*, 2004; Santoni *et al.*, 2008; Huang *et al.*, 2000). The co-crystal sates of acid···pyridine and acid···pyrimidine systems have been reported (Bhogala *et al.*, 2003; Du *et al.*, 2005; Hou *et al.*, 2008). Here we report the co-crystal states of L1 and L2.

The title molecular structure is shown in Fig. 1. The asymmetric unit contains 5-(4-pyridyl)pyrimidine molecule (L1) and a benzene-1,3,5-tricarboxylic acid molecule (L2). In the crystal, L1 and L2 arrange in an alternate disposition along the crystallographic *c*-axis. A H-bonding driven double chain was generated from O—H···N and O—H···O hydrogen bonds between these molecules (Fig. 2). The asymmetric hydrogen bonds influence the intramolecular coplanar of L2 and different dihedral angles of 7.92 (9), 8.68 (10) and 17.07 (10)° are formed between benzene ring and the carboxyl groups. Pyridine ring is twisted to pyrimidine ring at a dihedral angle of 33.26 (11)°, but nearly coplanar with benzene ring of L2 (the dihedral angle, 12.0 (8)°).

### Experimental

A CH_2_Cl_2_ and CH_3_CN solution (15 mL, 1:1, *v*/*v*) of 5-(4-pyridyl)pyrimidine molecule and benzene-1,3,5-tricarboxylic acid (21.0 mg, 0.1 mmol) was kept at room temperature. Upon slow evaporation of the solvent about 5 days, colorless crystals were obtained.

### Refinement

The carboxyl-H atoms were located in a difference Fourier map and refined isotropically. Aromatic H atoms were placed in idealized positions and treated as riding with C—H = 0.93 Å and *U*_iso_(H) = 1.2*U*_eq_(C).

### Crystal data

**Table:**

C_9_H_7_N_3_·C_9_H_6_O_6_	*F*(000) = 760
*M**_r_* = 367.31	*D*_x_ = 1.520 Mg m^−^^3^
Monoclinic, *P*2_1_/*c*	Mo *K*α radiation, λ = 0.71073 Å
Hall symbol: -P 2ybc	Cell parameters from 1381 reflections
*a* = 8.3532 (19) Å	θ = 2.5–22.7°
*b* = 14.865 (3) Å	µ = 0.12 mm^−^^1^
*c* = 13.066 (3) Å	*T* = 298 K
β = 98.325 (4)°	Block, colourless
*V* = 1605.4 (6) Å^3^	0.32 × 0.12 × 0.10 mm
*Z* = 4	

### Data collection

**Table:**

Bruker SMART APEX CCD diffractometer	2142 reflections with *I* > 2σ(*I*)
Radiation source: fine-focus sealed tube	*R*_int_ = 0.042
graphite	θ_max_ = 25.5°, θ_min_ = 2.1°
φ and ω scans	*h* = −10→10
8278 measured reflections	*k* = −18→17
2967 independent reflections	*l* = −9→15

### Refinement

**Table:**

Refinement on *F*^2^	Primary atom site location: structure-invariant direct methods
Least-squares matrix: full	Secondary atom site location: difference Fourier map
*R*[*F*^2^ > 2σ(*F*^2^)] = 0.055	Hydrogen site location: inferred from neighbouring sites
*wR*(*F*^2^) = 0.130	H atoms treated by a mixture of independent and constrained refinement
*S* = 1.04	*w* = 1/[σ^2^(*F*_o_^2^) + (0.0605*P*)^2^ + 0.0652*P*] where *P* = (*F*_o_^2^ + 2*F*_c_^2^)/3
2967 reflections	(Δ/σ)_max_ < 0.001
256 parameters	Δρ_max_ = 0.22 e Å^−^^3^
0 restraints	Δρ_min_ = −0.21 e Å^−^^3^

### Special details

**Table:**

Geometry. All esds (except the esd in the dihedral angle between two l.s. planes) are estimated using the full covariance matrix. The cell esds are taken into account individually in the estimation of esds in distances, angles and torsion angles; correlations between esds in cell parameters are only used when they are defined by crystal symmetry. An approximate (isotropic) treatment of cell esds is used for estimating esds involving l.s. planes.
Refinement. Refinement of F^2^ against ALL reflections. The weighted R-factor wR and goodness of fit S are based on F^2^, conventional R-factors R are based on F, with F set to zero for negative F^2^. The threshold expression of F^2^ > 2sigma(F^2^) is used only for calculating R-factors(gt) etc. and is not relevant to the choice of reflections for refinement. R-factors based on F^2^ are statistically about twice as large as those based on F, and R- factors based on ALL data will be even larger.

### Fractional atomic coordinates and isotropic or equivalent isotropic displacement parameters (Å^2^)

**Table:**

	*x*	*y*	*z*	*U*_iso_*/*U*_eq_	
C1	1.0045 (3)	0.23487 (15)	0.04299 (17)	0.0307 (5)	
C2	0.9253 (3)	0.28752 (14)	0.11876 (16)	0.0298 (5)	
C3	0.9233 (3)	0.38061 (15)	0.11112 (17)	0.0328 (6)	
H3	0.9717	0.4086	0.0598	0.039*	
C4	0.8495 (3)	0.43246 (14)	0.17958 (16)	0.0314 (6)	
C5	0.7764 (3)	0.38975 (15)	0.25547 (16)	0.0323 (6)	
H5	0.7254	0.4239	0.3009	0.039*	
C6	0.7787 (3)	0.29718 (15)	0.26421 (16)	0.0314 (5)	
C7	0.8537 (3)	0.24592 (15)	0.19591 (16)	0.0315 (6)	
H7	0.8559	0.1836	0.2018	0.038*	
C8	0.6958 (3)	0.24978 (16)	0.34319 (17)	0.0327 (6)	
C9	0.8432 (3)	0.53210 (16)	0.17124 (18)	0.0402 (6)	
C10	0.4491 (3)	0.13542 (17)	0.52900 (19)	0.0462 (7)	
H10	0.4782	0.1049	0.4723	0.055*	
C11	0.3919 (3)	0.08600 (16)	0.60484 (18)	0.0405 (7)	
H11	0.3804	0.0239	0.5985	0.049*	
C12	0.3513 (3)	0.13035 (15)	0.69125 (16)	0.0302 (5)	
C13	0.3600 (3)	0.22316 (15)	0.69197 (17)	0.0319 (6)	
H13	0.3274	0.2556	0.7462	0.038*	
C14	0.4171 (3)	0.26728 (16)	0.61198 (17)	0.0361 (6)	
H14	0.4224	0.3298	0.6137	0.043*	
C15	0.3122 (3)	0.07958 (14)	0.78172 (16)	0.0284 (5)	
C16	0.2130 (3)	0.11360 (15)	0.84858 (17)	0.0354 (6)	
H16	0.1628	0.1689	0.8337	0.043*	
C17	0.2625 (3)	−0.00820 (15)	0.95212 (18)	0.0423 (7)	
H17	0.2449	−0.0386	1.0117	0.051*	
C18	0.3827 (3)	−0.00268 (15)	0.80969 (18)	0.0402 (6)	
H18	0.4496	−0.0283	0.7666	0.048*	
N1	0.4653 (2)	0.22434 (14)	0.53205 (15)	0.0398 (5)	
N2	0.3604 (3)	−0.04710 (13)	0.89448 (16)	0.0470 (6)	
N3	0.1871 (2)	0.06964 (12)	0.93362 (14)	0.0380 (5)	
O1	1.0171 (2)	0.14904 (10)	0.06445 (13)	0.0432 (5)	
H1	1.071 (4)	0.1197 (19)	0.014 (2)	0.082 (10)*	
O2	1.0535 (2)	0.26844 (10)	−0.03140 (12)	0.0392 (5)	
O3	0.9472 (3)	0.56310 (13)	0.11264 (15)	0.0621 (7)	
H3A	0.930 (4)	0.622 (2)	0.095 (2)	0.088 (11)*	
O4	0.7531 (2)	0.57872 (11)	0.21148 (14)	0.0605 (6)	
O5	0.6348 (2)	0.30593 (11)	0.40541 (13)	0.0446 (5)	
H5A	0.572 (4)	0.276 (2)	0.449 (2)	0.093 (11)*	
O6	0.6847 (2)	0.16917 (11)	0.34744 (13)	0.0482 (5)	

### Atomic displacement parameters (Å^2^)

**Table:**

	*U*^11^	*U*^22^	*U*^33^	*U*^12^	*U*^13^	*U*^23^
C1	0.0310 (14)	0.0294 (13)	0.0331 (13)	0.0002 (10)	0.0093 (11)	−0.0013 (10)
C2	0.0296 (14)	0.0323 (13)	0.0289 (12)	−0.0002 (10)	0.0087 (10)	0.0011 (9)
C3	0.0368 (15)	0.0331 (13)	0.0315 (13)	−0.0010 (10)	0.0153 (11)	0.0037 (9)
C4	0.0343 (15)	0.0328 (13)	0.0285 (13)	0.0029 (10)	0.0096 (11)	0.0016 (9)
C5	0.0340 (14)	0.0378 (14)	0.0270 (12)	0.0016 (11)	0.0108 (11)	−0.0009 (10)
C6	0.0283 (14)	0.0376 (13)	0.0293 (13)	−0.0006 (10)	0.0079 (11)	−0.0004 (10)
C7	0.0337 (14)	0.0290 (12)	0.0330 (13)	−0.0006 (10)	0.0092 (11)	0.0006 (9)
C8	0.0343 (15)	0.0370 (14)	0.0280 (13)	−0.0007 (11)	0.0081 (11)	0.0006 (10)
C9	0.0546 (18)	0.0356 (14)	0.0332 (13)	0.0027 (12)	0.0161 (13)	0.0019 (11)
C10	0.0549 (19)	0.0501 (17)	0.0381 (15)	0.0041 (13)	0.0222 (14)	−0.0040 (12)
C11	0.0529 (18)	0.0337 (14)	0.0379 (14)	0.0006 (12)	0.0168 (13)	−0.0050 (10)
C12	0.0283 (14)	0.0316 (13)	0.0318 (13)	−0.0003 (10)	0.0079 (10)	−0.0017 (9)
C13	0.0343 (15)	0.0319 (13)	0.0311 (13)	0.0005 (10)	0.0107 (11)	0.0001 (10)
C14	0.0379 (15)	0.0329 (13)	0.0391 (14)	−0.0021 (11)	0.0110 (12)	0.0022 (10)
C15	0.0292 (14)	0.0245 (12)	0.0326 (13)	−0.0041 (10)	0.0085 (11)	−0.0022 (9)
C16	0.0399 (16)	0.0287 (12)	0.0404 (14)	0.0026 (10)	0.0149 (12)	0.0027 (10)
C17	0.0608 (19)	0.0314 (13)	0.0384 (14)	−0.0028 (12)	0.0197 (13)	0.0047 (11)
C18	0.0528 (18)	0.0288 (13)	0.0435 (15)	0.0004 (12)	0.0224 (13)	−0.0029 (11)
N1	0.0390 (13)	0.0460 (13)	0.0369 (12)	−0.0002 (10)	0.0142 (10)	0.0045 (9)
N2	0.0670 (17)	0.0312 (11)	0.0483 (13)	0.0081 (10)	0.0269 (12)	0.0056 (9)
N3	0.0478 (14)	0.0334 (11)	0.0365 (12)	0.0016 (10)	0.0192 (10)	0.0021 (9)
O1	0.0601 (13)	0.0291 (9)	0.0477 (11)	0.0043 (8)	0.0320 (10)	0.0012 (7)
O2	0.0541 (12)	0.0323 (9)	0.0365 (10)	0.0032 (8)	0.0249 (9)	0.0041 (7)
O3	0.0953 (17)	0.0323 (11)	0.0711 (14)	0.0013 (10)	0.0537 (13)	0.0090 (9)
O4	0.0866 (16)	0.0362 (10)	0.0679 (14)	0.0151 (10)	0.0422 (12)	−0.0003 (9)
O5	0.0600 (13)	0.0422 (10)	0.0386 (10)	−0.0023 (9)	0.0306 (10)	0.0002 (8)
O6	0.0666 (14)	0.0344 (10)	0.0498 (11)	−0.0034 (9)	0.0288 (10)	0.0039 (8)

### Geometric parameters (Å, °)

**Table:**

C1—O2	1.215 (2)	C11—C12	1.391 (3)
C1—O1	1.307 (3)	C11—H11	0.9300
C1—C2	1.490 (3)	C12—C13	1.381 (3)
C2—C3	1.387 (3)	C12—C15	1.478 (3)
C2—C7	1.390 (3)	C13—C14	1.377 (3)
C3—C4	1.390 (3)	C13—H13	0.9300
C3—H3	0.9300	C14—N1	1.335 (3)
C4—C5	1.391 (3)	C14—H14	0.9300
C4—C9	1.485 (3)	C15—C18	1.383 (3)
C5—C6	1.381 (3)	C15—C16	1.384 (3)
C5—H5	0.9300	C16—N3	1.333 (3)
C6—C7	1.389 (3)	C16—H16	0.9300
C6—C8	1.499 (3)	C17—N2	1.322 (3)
C7—H7	0.9300	C17—N3	1.323 (3)
C8—O6	1.204 (3)	C17—H17	0.9300
C8—O5	1.318 (3)	C18—N2	1.326 (3)
C9—O4	1.199 (3)	C18—H18	0.9300
C9—O3	1.321 (3)	O1—H1	0.95 (3)
C10—N1	1.329 (3)	O3—H3A	0.92 (3)
C10—C11	1.374 (3)	O5—H5A	0.95 (3)
C10—H10	0.9300		
			
O2—C1—O1	123.10 (19)	C10—C11—C12	118.9 (2)
O2—C1—C2	123.3 (2)	C10—C11—H11	120.6
O1—C1—C2	113.59 (18)	C12—C11—H11	120.6
C3—C2—C7	119.60 (19)	C13—C12—C11	117.41 (19)
C3—C2—C1	118.57 (18)	C13—C12—C15	121.45 (18)
C7—C2—C1	121.8 (2)	C11—C12—C15	121.0 (2)
C2—C3—C4	120.57 (19)	C14—C13—C12	119.6 (2)
C2—C3—H3	119.7	C14—C13—H13	120.2
C4—C3—H3	119.7	C12—C13—H13	120.2
C3—C4—C5	119.1 (2)	N1—C14—C13	122.9 (2)
C3—C4—C9	121.33 (19)	N1—C14—H14	118.5
C5—C4—C9	119.52 (19)	C13—C14—H14	118.5
C6—C5—C4	120.8 (2)	C18—C15—C16	115.3 (2)
C6—C5—H5	119.6	C18—C15—C12	121.86 (19)
C4—C5—H5	119.6	C16—C15—C12	122.7 (2)
C5—C6—C7	119.71 (19)	N3—C16—C15	122.1 (2)
C5—C6—C8	121.53 (19)	N3—C16—H16	119.0
C7—C6—C8	118.7 (2)	C15—C16—H16	119.0
C6—C7—C2	120.2 (2)	N2—C17—N3	126.6 (2)
C6—C7—H7	119.9	N2—C17—H17	116.7
C2—C7—H7	119.9	N3—C17—H17	116.7
O6—C8—O5	124.2 (2)	N2—C18—C15	123.7 (2)
O6—C8—C6	123.10 (19)	N2—C18—H18	118.1
O5—C8—C6	112.7 (2)	C15—C18—H18	118.1
O4—C9—O3	124.0 (2)	C10—N1—C14	117.28 (19)
O4—C9—C4	124.2 (2)	C17—N2—C18	115.6 (2)
O3—C9—C4	111.8 (2)	C17—N3—C16	116.78 (19)
N1—C10—C11	123.7 (2)	C1—O1—H1	109.3 (17)
N1—C10—H10	118.1	C9—O3—H3A	113.0 (19)
C11—C10—H10	118.1	C8—O5—H5A	111.9 (18)
			
O2—C1—C2—C3	−7.5 (3)	C3—C4—C9—O3	−16.2 (3)
O1—C1—C2—C3	172.1 (2)	C5—C4—C9—O3	165.7 (2)
O2—C1—C2—C7	172.3 (2)	N1—C10—C11—C12	−1.5 (4)
O1—C1—C2—C7	−8.1 (3)	C10—C11—C12—C13	4.7 (4)
C7—C2—C3—C4	−0.3 (4)	C10—C11—C12—C15	−171.0 (2)
C1—C2—C3—C4	179.4 (2)	C11—C12—C13—C14	−4.1 (3)
C2—C3—C4—C5	−0.5 (3)	C15—C12—C13—C14	171.5 (2)
C2—C3—C4—C9	−178.7 (2)	C12—C13—C14—N1	0.1 (4)
C3—C4—C5—C6	1.0 (3)	C13—C12—C15—C18	−144.0 (2)
C9—C4—C5—C6	179.2 (2)	C11—C12—C15—C18	31.5 (3)
C4—C5—C6—C7	−0.6 (3)	C13—C12—C15—C16	31.0 (3)
C4—C5—C6—C8	−177.9 (2)	C11—C12—C15—C16	−153.5 (2)
C5—C6—C7—C2	−0.3 (3)	C18—C15—C16—N3	−0.3 (4)
C8—C6—C7—C2	177.1 (2)	C12—C15—C16—N3	−175.5 (2)
C3—C2—C7—C6	0.8 (3)	C16—C15—C18—N2	−0.6 (4)
C1—C2—C7—C6	−179.0 (2)	C12—C15—C18—N2	174.7 (2)
C5—C6—C8—O6	173.9 (2)	C11—C10—N1—C14	−2.5 (4)
C7—C6—C8—O6	−3.5 (4)	C13—C14—N1—C10	3.2 (4)
C5—C6—C8—O5	−4.9 (3)	N3—C17—N2—C18	−0.4 (4)
C7—C6—C8—O5	177.7 (2)	C15—C18—N2—C17	0.9 (4)
C3—C4—C9—O4	162.8 (3)	N2—C17—N3—C16	−0.4 (4)
C5—C4—C9—O4	−15.4 (4)	C15—C16—N3—C17	0.7 (4)

### Hydrogen-bond geometry (Å, °)

**Table:**

*D*—H···*A*	*D*—H	H···*A*	*D*···*A*	*D*—H···*A*
O1—H1···N3^i^	0.95 (3)	1.70 (3)	2.652 (2)	173 (3)
O3—H3A···O2^ii^	0.92 (3)	1.84 (3)	2.720 (2)	161 (3)
O5—H5A···N1	0.95 (3)	1.68 (3)	2.626 (2)	177 (3)
C3—H3···O3^ii^	0.93	2.48	3.367 (3)	159
C14—H14···N2^iii^	0.93	2.59	3.335 (3)	137

Symmetry codes: (i) *x*+1, *y*, *z*−1; (ii) −*x*+2, −*y*+1, −*z*; (iii) −*x*+1, *y*+1/2, −*z*+3/2.
